# Likelihood of malignancy in thyroid nodules according to a proposed Thyroid Imaging Reporting and Data System (TI-RADS) classification merging suspicious and benign ultrasound features

**DOI:** 10.1590/2359-3997000000262

**Published:** 2017-03-20

**Authors:** Ricardo Luiz Costantin Delfim, Leticia Carrasco Garcez da Veiga, Ana Paula Aguiar Vidal, Flávia Paiva Proença Lobo Lopes, Mário Vaisman, Patrícia de Fatima dos Santos Teixeira

**Affiliations:** 1 Departamento de Endocrinologia Universidade Federal do Rio de Janeiro Rio de Janeiro RJ Brasil Departamento de Endocrinologia, Universidade Federal do Rio de Janeiro (UFRJ). Clínica de Diagnóstico por Imagem (CDPI), Rio de Janeiro, RJ, Brasil; 2 Departamento de Endocrinologia Universidade Federal do Rio de Janeiro Rio de Janeiro RJ Brasil Departamento de Endocrinologia, Universidade Federal do Rio de Janeiro (UFRJ), Rio de Janeiro, RJ, Brasil; 3 Departamento de Radiologia Universidade Federal do Rio de Janeiro Rio de Janeiro RJ Brasil Departamento de Radiologia, Universidade Federal do Rio de Janeiro (UFRJ), Clínica de Diagnóstico por Imagem (CDPI), Rio de Janeiro, RJ, Brasil

**Keywords:** Thyroid nodules, TI-RADS, thyroid cancer

## Abstract

**Objective:**

The aim of this study was to describe the ultrasound features of benign and malignant thyroid nodules and evaluate the likelihood of malignancy associated with each feature according to the Bethesda System for Reporting Thyroid Cytopathology and histopathology. With this analysis, we propose a new TI-RADS classification system.

**Materials and methods:**

The likelihood of malignancy from ultrasound features were assessed in 1413 thyroid nodules according to the Bethesda System for Reporting Thyroid Cytopathology and histopathological findings. A score was established by attributing different weights to each ultrasound feature evaluated.

**Results:**

Features positively associated with malignancy in bivariate analysis received a score weight of +1. We attributed a weight of +2 to features which were independently associated with malignancy in a multivariate analysis and +3 for those associated with the highest odds ratio for malignancy (> 10.0). Hence, hypoechogenicity (graded as mild, moderate or marked, according to a comparison with the overlying strap muscle), microcalcification and irregular/microlobulated margin received the highest weights in our scoring system. Features that were negatively associated with malignancy received weights of -2 or -1. In the proposed system a cutoff score of 2 (sensitivity 97.4% and specificity 51.6%) was adopted as a transition between probably benign (TI-RADS 3) and TI-RADS 4a nodules. Overall, the frequency of malignancy in thyroid nodules according to the categories was 1.0% for TI-RADS 3, 7.8% for TI-RADS 4a, 35.3% for TI-RADS 4b, and 84.7% for TI-RADS 5.

**Conclusion:**

A newly proposed TI-RADS classification adequately assessed the likelihood of malignancy in thyroid nodules.

## INTRODUCTION

The incidence of thyroid nodules has increased 2–4-fold over the past three decades, mainly due to increased use of ultrasound and advancement in ultrasound technology (1,2). According to recent guidelines and recommendations reported by different scientific societies (3-6), ultrasound remains the most important tool in the initial evaluation of thyroid nodules since it has the ability to detect and diagnose potentially malignant thyroid nodules.

Several authors (7-12) have proposed different Thyroid Imaging Reporting and Data System (TI-RADS) classifications to standardize thyroid ultrasound reports, as demonstrated with the Breast Imaging Reporting and Data System (BI-RADS^®^) (13). Researchers have recently attempted to validate this type of approach as an instrument for use in clinical practice, with some authors proposing different TI-RADS versions in selected populations (14-20).

The first study proposing a TI-RADS classification was published by Horvath and cols. correlating 10 ultrasound patterns with the risk of malignancy in thyroid nodules (7). The study focused on relevant patterns in thyroid nodules with a low likelihood of malignancy and described important features related with benignity. Thereafter, a different classification and scoring system was proposed (8) using binary logistic regression to assess different odds ratios (OR) for each suspicious feature and generate an equation leading to a final score. The TI-RADS proposed by Kwak and cols. (9) was based on a practical and simplified scoring system to identify suspicious findings; each feature received identical weight in the proposed score, and higher scores were attributed to the occurrence of more than one suspicious ultrasound feature in the same nodule.

Russ and cols. (10) proposed in the form of an atlas a classification of thyroid nodules using seven different ultrasound patterns and creating their own TI-RADS categories. A simplified version of this classification, which excluded from the assessment Doppler and elastography, was subsequently created (11). Later, Russ and cols. (12) validated their own proposed classification in 4550 nodules, which was further validated in other 242 nodules (17).

In order to improve in own previous classification Kwak and cols. (21) conducted a multicenter study to develop a score attributing different values to each suspicious feature to the final score. In this proposed classification, the authors did not include benign features (21).

Until now, none of the proposed TI-RADS classifications has been universally accepted. The latest guidelines on thyroid nodules and differentiated thyroid cancer developed by the ATA (4), proposes a risk classification based on different ultrasound patterns categorized into five groups. In this classification, the risk of malignancy in thyroid nodules increases from < 3% (very low suspicion) to > 70-90% (high suspicion). According to this classification, hypoechoic nodules considered as highly suspicious also display other suspicious features, such as microcalcification or irregular/microlobulated margin. This proposed approach, which is based on groups of ultrasound patterns, facilitates the clinical management of thyroid nodules. However, some nodules do not fall into any of the five proposed pattern groups in the ATA classification (4) (*e.g.*, isoechoic nodules with micro- or macrocalcification). This fact may explain the gap seen in the risk of malignancy, from 20% in thyroid nodules with intermediate ultrasound patterns to 70% in those with a highly suspicious ultrasound pattern. A similar gap has also been reported in the guidelines proposed by the AACE/ACE/AME (5), which included three classes of ultrasound patterns categorized according to risk of malignancy into high, intermediate, and low.

The American College of Radiology recently assembled a committee to initiate a process to develop their own TI-RADS. The first step of the committee was to create the Thyroid Ultrasound Reporting Lexicon to describe ultrasound characteristics of thyroid nodules, providing concise written definitions and illustrations to guide practitioners (22). Also recently, the Korean Society of Thyroid Radiology proposed a modification to the TI-RADS system (K-TIRADS) using a flowchart-guided classification according to the presence or absence of different ultrasound features found in thyroid nodules (6).

The aim of this study was to describe the ultrasound features of benign and malignant thyroid nodules and evaluate the likelihood of malignancy associated with each feature according to the Bethesda System for Reporting Thyroid Cytopathology (23) and histopathology. With this analysis, we propose a new TI-RADS classification system.

## MATERIALS AND METHODS

### Study design and population

We conducted a retrospective, case-control study to analyze the ultrasound features of 1413 thyroid nodules evaluated with FNAB between January 2008 and June 2013 at two institutions (*CDPI – Clínica de Diagnóstico por Imagem* and *Labs D’or*, both in Rio de Janeiro, Brazil). The criteria for the selection of the thyroid nodules were based on cytopathological features. All cytopathological samples obtained by FNAB were examined according to the Bethesda classification (23). The selected cases included thyroid nodules exhibiting suspicious or malignant cytopathology (category V or VI), which were then surgically resected and had a confirmatory histopathological report. The control sample included thyroid nodules with a benign cytopathology (category II). Most patients in the control group were followed up, and 6.5% of their nodules were evaluated with a second FNAB with a concordant cytopathology, confirming their benign nature (4,24). A benign status was also established by histopathological assessment in 2.0% of the control nodules. Nodules confirmed as benign were included in a subanalysis; those with confirmatory histopathology or a second FNAB were used as controls and compared with malignant nodules (cases). Nodules presenting any pathological divergence were excluded.

All patients had been referred for FNAB or surgery by their own physicians in an outpatient clinical setting. A minimal nodular size for enrollment in the study was not established. The study, which did not have an interventional design, was approved by the local ethics committee (053560/2012). In addition, all patients signed an informed consent form after receiving a clear explanation of the FNAB procedure, limitations, and possible complications.

### Thyroid ultrasound and FNAB evaluations

Both ultrasound and FNAB were performed by the same expert radiologist with an experience of over 25 years performing ultrasound and more than 15 years performing FNAB. The ultrasound examinations were performed using different 6–15 MHz linear-array probes and one of the following equipment: HDI 5000 Ultrasound System (Philips Medical System, Bothell, WA, USA), Xario SSA-660A (Toshiba Medical System Corporation), a Logiq 5 Expert (GE Medical System, Milwaukee, WI, USA), or a Logiq E9 (GE Medical System, Milwaukee, WI, USA).

After a short interview, the patients underwent a thyroid ultrasound examination followed by FNAB. All procedures were performed under real-time visualization, without an aspirator and with a similar freehand biopsy technique, independent of the institution in which the examination was performed. The ultrasound features of each lesion were meticulously classified immediately after the examination. All cytopathology reports issued prior to the Bethesda report (23) were reviewed by a single pathologist who issued a report based on the new classification system.

A random subsample of 5% of the ultrasound recordings was also evaluated by an external researcher with expertise in ultrasound, without prior knowledge of cytopathological reports. A high agreement was observed between the two researchers (kappa = 0.99, p < 0.001).

All nodules were evaluated and classified according to the presence of 20 predefined ultrasound features, mostly retrieved from a literature review (4,5,7-11,21,25-34) and detailed in Table 1. These features were included in a multiple logistic regression analysis to determine whether they were (or not) independently associated with the likelihood of malignancy (4,5,8-11,24-26,28-30,34). Among all ultrasound features, 13 were likely to be associated with malignancy, as previously described: (i) solid appearance, (ii) hypoechogenicity (any degree [graded as mild, moderate or marked]), according to a comparison with the overlying strap muscle), (iii) moderate to marked hypoechogenicity, (iv) marked hypoechogenicity, (v) presence of peripheral and/or inner microcalcification, (vi) absence of a halo, (vii) irregular thick halo, (viii) irregular/microlobulated margin, (ix) blurred margin, (x) non-ovoid shape, (xi) taller-than-wide shape, (xii) presence of any degree of central blood flow, and (xiii) predominantly central blood flow (*i.e.*, central blood flow alone or more accentuated than the peripheral one). Conversely, the following five ultrasound features were considered to be potentially associated with benign nodules (4,5,8,25,27,29,31,32): (i) a spongiform appearance, (ii) hyperechogenicity, (iii) eggshell calcification, (iv) presence of colloid crystal, and (v) thin regular halo. Indeterminate features for likelihood associations (based on disagreements in the literature) assessed and included in the analysis were (i) peripheral and/or inner macrocalcification and (ii) hyperechoic spot (5,7,8-10,29,31).

**Table 1:** Standardized definition for ultrasound features of thyroid nodules

Ultrasound features		Definition
Composition	Solid appearance	> 90% of nodule component is solid (24)
	Spongiform appearance	Predominantly cystic with multiple degenerative areas (> 50% in its composition) (25)
Grade of echogenicity of the solid component	Hyperechogenicity	Echogenicity greater than thyroid parenchyma (10,11,25,27)
	Hypoechogenicity (any degree)	Echogenicity lesser than thyroid parenchyma (28,29), including thyroid nodules with mild*, moderate** and marked *** hypoechogenicity (30)
	Moderate to marked hypoechogenicity	Echogenicity similar and lesser than that of strap muscle, including thyroid nodules with moderate** and marked*** hypoechogenicity
	Marked hypoechogenicity	Echogenicity lesser than that of strap muscle (30)
Margins and halos	Absence of a halo	No identified hypoechogenic halo
	Irregular thick halo	Irregular halo, ≥ 2 mm in thickness (26)
	Regular thin halo	Complete and regular, < 2 mm in thickness (26)
	Irregular/microlobulated margin	Irregular or microlobulated margins (8,9,21,30)
	Blurred margin	Not well defined margin (31)
Presence of different hyperechogenic spots, including any kind of calcifications	Microcalcification	Peripheral and/or inner microcalcification, defined by hyperechogenic spot ≤ 2 mm, either with or without acoustic shadow (26)
	Macrocalcification	Peripheral and/or inner hyperechogenic coarse or spot > 2 mm, either with or without acoustic shadow (26)
	Egg shell calcification	Complete and regular calcification border (5,8,25)
	Colloid Crystal	Hyperechogenic spot with comet-tail artifact (birefringence) (29,32)
	Unspecific hyperechoic spots	Hyperechogenic spot without acoustic shadow or comet-tail artifact that is not well characterized as calcification or colloid crystal (29,31)
Shape	Non-ovoid shape	Anteroposterior diameter greater than its transverse or longitudinal one
	Taller-than-wide shape	Anteroposterior diameter greater than its transverse diameter (30)
Doppler color flow	Any degree of central flow	Any degree of central flow (28,33)
	Predominant central flow	Central flow greater than peripheral blood flow and exclusively central flow (34)

* Echogenicity lesser than thyroid parenchyma but greater than of strap muscle (10,12);

** Echogenicity similar to the strap muscle; *** echogenic lesser than of strap muscle, characterizing a marked hypoechogenicity (30).

### Statistical analysis

We performed all statistical analyses using the Statistical Package for the Social Sciences (SPSS) for Windows, version 17.0 (IBM). Continuous variables are presented as mean ± standard deviation (SD) (median). We compared these variables between two groups using the Mann-Whitney test. For comparisons among three or more groups, we used the Kruskal-Wallis test. We expressed categorical variables as percentages and compared these variables using the chi-squared test (c^2^) or Fisher’s exact test in bivariate analysis. Binary logistic regression was applied to determine in a multivariate analysis which specific covariates (ultrasound features) were independently associated with malignancy.

## RESULTS

We evaluated 1413 thyroid nodules, of which 1174 (83.1%) were classified as category II, 155 (11.0%) as category V, and 84 (5.9%) as category VI according to the Bethesda classification criteria. Overall, 1251 (88.5%) nodules were in women. There was no statistically significant difference between the Bethesda classification and gender (categories II: 89.1%; V: 86.5%; and VI: 84.7%; p = 0.307). Patients with a Bethesda II classification were significantly older (mean ages in each category: II, 52 years; V, 44 years; and VI, 46 years; p < 0.001).

We obtained a histopathological analysis of all thyroid nodules with a malignant or suspicious cytopathology (n = 239). We observed a high diagnostic agreement between the cytopathological and histopathological diagnoses (kappa = 0.96; p < 0.001). The histopathological examination confirmed malignancy in 98.7% (153/155) and 98.8% (83/84) of the nodules categorized as V and VI, respectively. Among the benign nodules, a confirmatory diagnosis was obtained in a subgroup of the sample (n = 99; 8.4%) by histopathology (n = 23) or a second FNAB (n = 76).

### Associations between ultrasound features and Bethesda cytopathology results

**Figure 1:**
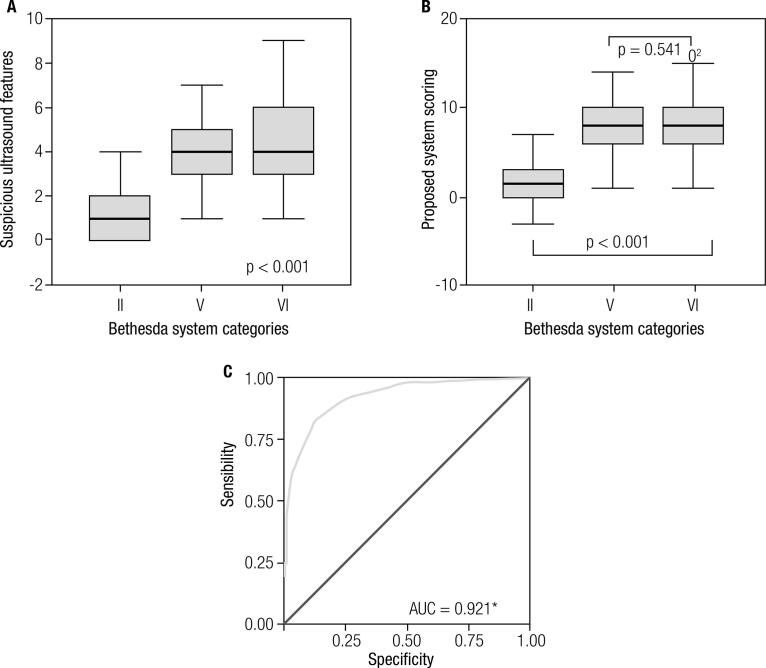
Blox pot graphs and receiver operating characteristics (ROC). A. Distribution of suspicious ultrasound features by Bethesda system categories. B. Distribution of proposed system scoring by Bethesda system categories. C. ROC curve was applied to determine the best cut off with high sensitivity and specificity for the highest risk categories of maligancy in the proposed score system. * AUC: area under the curve.

Suspicious ultrasound features increased in frequency along with the degree of suspicion on cytopathology (Table 2) and the number of suspicious ultrasound features presented in the thyroid nodules was higher according to the likelihood of malignancy identified on cytopathology (Figure 1). The numbers of suspicious features were 3.7 ± 1.3 and 3.3 ± 1.2 in Bethesda VI and V nodules, respectively. These values were higher (p < 0.001) than those found in Bethesda II nodules (1.06 ± 1.4). The bivariate analysis revealed an association between each ultrasound feature and the likelihood of suspicious/malignant cytopathology (Table 2). The likelihood of confirmed malignancy, obtained by evaluating a subgroup of nodules with a confirmed diagnosis of malignancy, is also presented in Table 2. Eggshell calcification was not detected in any of the thyroid nodules removed by surgery. Table 2 also lists the results of the multivariate analysis, showing features independently associated with reported endpoints (*i.e.*, “suspicious/malignant cytopathology” or “confirmed malignancy”). In a subanalysis including thyroid nodules with a confirmed diagnosis, the same ultrasound features were associated with either an increased or reduced likelihood of malignancy. However, five of the features (*i.e.*, blurred margin, thick irregular halo, colloid crystal, hyperechoic spot, and macrocalcification) were no longer statistically significant.

**Table 2:** Statistical analysis

Ultrasound features	Frequency distribution of ultrasound features according to the Bethesda system (%)				Relationship each ultrasound feature with cytopathological and histopathological assessments							

	Bethesda system categories #				Benign and suspicious/malignant cytopathology (n = 1413)				Subgroup of nodules with histopathological assessment (n = 338)			

	II	V	VI	p value	Bivariate analysis*	p value	Multivariate analysis*	p value	Bivariate analysis*	p value	Multivariate analysis*	p value
Solid appearance	48.5 (n = 567)	92.3 (n = 143)	92.0 (n = 77)	**< 0.001**	**12.3 (7.6-19.9)**	**< 0.001**	**5.14 (2.8-9.4)**	**< 0.001**	**10.2 (4.5-18.9)**	**< 0.001**	**3.9 (1.5-9.7)**	**0.04**
Hypoechogenicity (any degree)	34.2 (n = 401)	93.5 (n = 145)	93.0 (n = 78)	**< 0.001**	**26.8 (15.8-45.0)**	**< 0.001**	**4.7 (2.4-9.4)**	**< 0.001**	**22.3 (11.7-42.8)**	**< 0.001**	**4.3 (1.7-11.1)**	**0.02**
Marked hypoechogenicity	12.9 (n = 149)	63.0 (n = 97)	73.5 (n = 61)	**< 0.001**	**8.8 (5.8-13.5)**	**< 0.001**			**9.9 (3.0-32.6)**	**< 0.001**		
Moderate to marked hypoechogenicity	3.7 (n = 43)	26.5 (n = 41)	22.6 (n = 19)	**< 0.001**	**13.5 (9.8-18.6)**	**< 0.001**	**3.45 (2.0-9.0)**	**< 0.001**	**13.2 (6.9-25.2)**	**< 0.001**	**3.43 (1.3-9.2)**	**0.01**
Irregular/microlobulated margin	0.7 (n = 8)	34.8 (n = 54)	40.5 (n = 34)	**< 0.001**	**84.5 (40.2-178)**	**< 0.001**	**28.8 (10.9-75.9)**	**< 0.001**	**56.1 (7.7-409)**	**0.001**		
Blurred margin	11.7 (n = 136)	16.6 (n = 25)	27.4 (n = 23)	**< 0.001**	**1.9 (1.3-2.8)**	**< 0.001**	**0.38 (0.2-0.8)**	**0.009**	1.8 (0.9-3.76)	0.147		
Absence of a halo	77.5 (n = 504)	90.9 (n = 140)	86.9 (n = 73)	**< 0.001**	**2.5 (1.6-3.8)**	**< 0,001**			**3.2 (1.7-5.9)**	**< 0.001**		
Irregular/thick halo	0.6 (n = 7)	2.6 (n = 4)	1.2 (n = 1)	0.09	**3.5 (1.0-11.3)**	**0.05**			1.0 (1.0-1.01)	0.628		
Microcalcification	2.1 (n = 25)	39.0 (n = 60)	52.4 (n = 44)	**< 0.001**	**35.6 (22.2-57.1)**	**< 0.001**	**12.6 (6.3-25.2)**	**< 0.001**	**24.8 (17.6-80.6)**	**0.001**	**12.0 (2.6 -54.5)**	**0.01**
Taller-than-wide	8.7 (n = 101)	8.0 (n = 14)	19.8 (n = 18)	**0.020**	1.5 (0.9-2.4)	0.58			2.3 (0.9-5.7)	0.07		
Non-ovoid shape	9.0 (n = 104)	9.0 (n = 14)	18.1 (n = 15)	**0.026**	1.4 (0.9-2.2)	0.06			**4.1 (1.2-13.8)**	**0.015**		
Any degree of central flow	71,1 (n = 820)	66.0 (n = 95)	72.3 (n = 60)	0.424	0.9 (0.6-1.2)	0.40			0.7 (0.3-1.1)	0.10		
Predominantly central flow	3.2 (n = 37)	14.2 (n = 22)	11.9 (n = 10)	**0.001**	**4.7 (2.9-7.8)**	**< 0.001**	**2.28 (1.2-5.1)**	**0.045**	**3.4 (1.2-9.9)**	**0.02**		
Spongiform appearance	2.0 (n = 24)	0	0	0.083	**0.98 (0.97-0.98)**	**0.001**			**0.98 (0.95-1.0)**	**0.08**		
Eggshell calcification	0.2 (n = 2)	0	0	0.467	0.99 (0.99-1.0)	0.648			N.E.**	N.E.**		
Colloid Crystal	2.3 (n = 27)	0	0	0.062	**0.97 (0.96-0.99)**	**0.01**			0.41 (0.03-6.7)	0.253		
Hyperechogenicity	2.6 (n = 30)	0	0	**0.044**	**0.97 (0.96-0.98)**	**0.04**			**0.98 (0.95-1.0)**	**0.08**		
Thin regular halo	21.9 (n = 256)	7.1 (n = 11)	11.9 (n = 10)	**< 0.001**	**0.34 (0.2-0.55)**	**< 0.001**			**0.25 (0.13-0.49)**	**0.001**		
Hyperechoic spot	7.2 (n = 84)	12.9 (n = 20)	9.5 (n = 8)	0.057	**1.7 (1.1-2.7)**	**0.03**	**4.01 (2.2-7.4)**	**< 0.001**	1.22 (0.5-2.7)	0.388	**3.1 (1.1-8.3)**	**0.03**
Macrocalcification	4.2 (n = 49)	14.2 (n = 22)	16.7 (n = 14)	**< 0.001**	**4.0 (2.6-6.4)**	**0.001**			2.2 (0.9-5.1)	0.122		

# Bethesda system categories included in analysis. * Odds ratio (confidence interval). ** N.E.: Not evaluated.

The blurred margin was the sole feature independently and negatively associated with malignancy (Table 2). Albeit, none of the spongiform nodules were malignant, this feature was not independently and negatively associated with the likelihood of malignancy (Table 2).

### TI-RADS scoring

We developed a scoring system based on the logistic multiple regression analysis and different weights assigned to each feature according to their association with the likelihood of malignancy on cytopathology, thoroughly detailed in Table 3. Features that were positively but not independently associated with a likelihood of malignancy received a weight of +1; these features included macrocalcification, non-ovoid shape, absence of a halo, and thick irregular halo. Features independently associated with a likelihood of malignancy that received a weight of +2 included a solid appearance, predominantly central flow, hyperechoic spot, hypoechogenicity (any degree), and moderate to marked hypoechogenicity. The presence of microcalcification and an irregular/microlobulated margin received a weight of +3 since their OR were the highest (> 10.0) compared with those of other features. Blurred margin, a feature independently associated with a benign status, received a weight of -2. Features that were associated with a benign status (but which the association did not emerge as independent in multivariate analysis) included a spongiform appearance, colloid crystal, hyperechogenicity, and a thin and regular halo. These last four features received a weight of -1 in our scoring system.

**Table 3:** Weight conception process for ultrasound features scoring

Ultrasound features	Weight conception process	Score
Marked hypoechogenicity	This feature alone was not independently associated with a likelihood of malignancy in multivariate analysis and received initially a score weight of +1. However, the weight of this feature increased since it is also included in the feature of hypoechogenicity of any degree (+2) and in moderate to marked hypoechogenicity (+2). The sum of all these weights resulted in the value of +5, attributed here	+5
Moderate hypoechogenicity	The presence of moderate to marked hypoechogenicity was independently associated with the likelihood of malignancy and received a weight of +2. However, the weight of this feature increased it is also included in the feature of hypoechogenicity of any degree (+2), yielding a score weight of +4. Moderate to marked hypoechogenicity term was replaced to “moderate hypoechogenicity”, as marked hypoechogenicity has its own score	+4
Microcalcification	Independently associated with the likelihood of malignancy (OR > 10.0)	+3
Irregular/microlobu lated margin	Independently associated with the likelihood of malignancy (OR > 10.0)	+3
Mild hypoechogenicity	This degree of hypoechogenicity received a weight based only on the feature of hypoechogenicity of any degree which did not meet the criteria for moderate or marked hypoechogenicity; it was then attributed a weight of +2 since it was included in the overall group of any degree hypoechoic nodules	+2
Solid appearance	Independently associated with the likelihood of malignancy (OR > 1.0 and ≤ 10.0)	+2
Undefined hyperechoic spot	Independently associated with the likelihood of malignancy (OR > 1.0 and ≤ 10.0)	+2
Predominantly central flow	Independently associated with the likelihood of malignancy (OR > 1.0 and ≤ 10.0)	+2
Non-ovoid shape	Ultrasound features positively associated with the likelihood of malignancy in bivariate but not multivariate analysis	+1
Macrocalcification		+1
Absence of a halo		+1
Irregular/thick halo		+1
Regular thin halo	Ultrasound features negatively associated with the likelihood of malignancy in bivariate but not multivariate analysis	-1
Crystal colloid		-1
Hyperechogenicity		-1
Spongiform appearance		-1
Blurred margin	Negatively and independently associated with the likelihood of malignancy	-2

OR: odds ratio.

**Figure 2:**
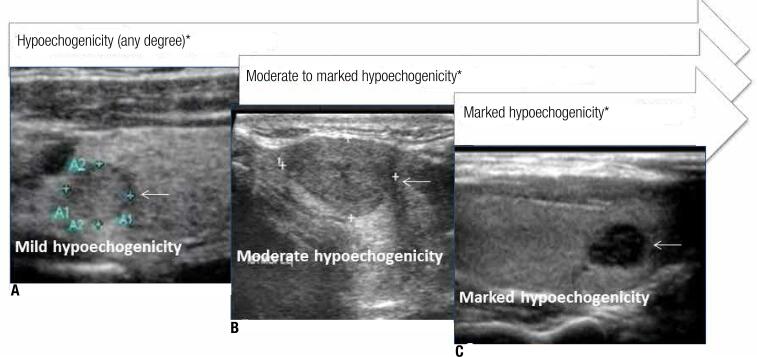
Hypoechogenicity gradation in thyroid nodules. Ultrasound images exemplify nodules that exhibit three grades of hypoechogenicity: (arrows). A. Mild hypoechogenicity: nodule presents echogenicity lesser than thyroid parenchyma and greater than the strap muscle (arrow). B. Moderate hypoechogenicity: nodule presents echogenicity similar to the strap muscle (arrow). C. Marked hypoechogenicity: nodule presents echogenicity lesser than the strap muscle (arrow). * Included in bivariate and multivariate analysis.

In terms of different grades of hypoechogenicity (Figure 2), we detected that nodules with much lower echogenicity had higher scores in our proposed scoring system. Marked hypoechogenicity was comprised in categories of thyroid nodules that presented hypoechogenicity of any degree (+2) and also in categories of moderate to marked hypoechogenicity (+2), besides the addition +1 (initial own score), total was +5 for these findings. Moderate hypoechogenicity was included in any degree hypoechogenicity (+2) plus the score of moderate to marked hypoechogenicity (+2), total +4 score. Mild hypoechogenicity, was assigned a final score +2 because it was not comprised neither moderate hypoechogenicity nor marked hypoechogenicity. This conceiving process is showed on Table 3.

Overall, the scores assigned to Bethesda category V and VI nodules were higher than those assigned to category II nodules (2.6 ± 2.5 [2.0] vs. 8.8 ± 3.18 [9.0]; p < 0.001) (Figure 1).

The Receiver Operating Characteristic (ROC) curve (Figure 1) yielded an area under the curve of 0.921 (CI 95%): 0.901–0.941) and demonstrated that a score of 5 reflected the best combined sensitivity (82.0%) and specificity (87.6%), as the cutoff point between the categories of low suspicion (TI-RADS 4a) and moderate to high suspicion for malignancy. The selected cutoff score that separated the category of highly suggestive of malignancy (TI-RADS 5) from low/moderate categories (TI-RADS 4b) was 9, which was the median score obtained for Bethesda category V and VI nodules (Figure 1). On the other hand, nodules scoring 2 were classified as probably benign; this was selected as the cutoff score between TI-RADS 3 (probably benign) and TI-RADS 4a (low suspicion), as shown in Table 4, and represents a value with high sensitivity (97.4%) but reduced specificity (51.6%), as shown in (Figure 1). Overall, the frequency of malignancy in thyroid nodules according to the categories was 1.0% for TI-RADS 3, 7.8% for TI-RADS 4a, 35.3% for TI-RADS 4b, and 84.7% for TI-RADS 5. By adopting these proposed criteria for our proposed TI-RADS, the frequency of malignant or suspicious cytopathology becomes very similar to that reported by the American College of Radiology for BI-RADS and prior Thyroid Imaging Reporting and Data System researches (7-13).

**Table 4:** Propose TI-RADS categories

TI-RADS 1: Negative
TI-RADS 2: Benign*
TI-RADS 3 (final score ≤ 2): Probably benign
TI-RADS 4a (final score 3–5): Low suspicion for malignancy
TI-RADS 4b (final score 6–9): Moderate suspicion for malignancy
TI-RADS 5 (final scores ≥ 10): Highly suggestive of malignancy

Suggestion 1: Investigate initially nodules ≥ 10 mm categorized as TI-RADS 4a. For those ≥ 5–10 mm, in the highest category, consider the patient’s decision before starting to investigate the nodule. Since, a follow-up is acceptable until the nodule achieves 10 mm, when it will then require investigation (3,4)

Suggestion 2: Investigate nodules with associated abnormal lymph nodes or potentially aggressive signs (paratracheal nodules, subcapsular location, or local invasion)

Suggestion 3: Consider a nodule in the next superior category if its growth rate or the patient’s personal/family history suggests a high risk of malignancy (3-6,10,18)

Suggestion 4: Consider the solid part of predominantly cystic nodules with an eccentric solid area as being a solid nodule and apply the score

* Simple cyst (purely anechoic content with thin, regular wall), in spite of this kind of nodule was not analyzed in our sample, it is the only one related to benignity, without any need to continue diagnostic investigation.

## DISCUSSION

In this study, we observed an association between categories of a newly proposed TI-RADS and the likelihood of malignancy. This finding is similar to that reported for the well-established BI-RADS concerning breast cancer. Additionally, our results are comparable to other TI-RADS classifications and are in accordance with recent guidelines classifications (4-6). Our study has quantified the ultrasound features in thyroid nodules by giving different weights to each feature positively or negatively associated with the likelihood of malignancy.

We found that all nodules with echogenicity lower than or similar to that of the overlying strap muscles were independently associated with malignancy. However, those thyroid nodules with marked hypoechogenicity received higher scores in our proposed scoring system. Due to that, we divided the feature of hypoechogenicity into degrees and found that marked hypoechogenicity played an important role in our proposed scoring system (Figure 2). Comparisons between the echogenicity of the nodule with that of the overlying strap muscles can improve cancer detection, especially in the context of thyroiditis, in which the thyroid parenchyma exhibits reduced echogenicity.

In support of our results, the presence of calcifications has been found to increase the likelihood of malignancy in different studies (29), particularly the presence of microcalcification. Since the size of microcalcifications has been reported to range from 0.5–3.0 mm in different studies (8-11,21;25,26,30), one should expect an overlap between micro- and macrocalcifications. However, macrocalcification as a possible suspicious feature has not been included in previous TI-RADS classifications (7,9,11,12,17). It is important to note that presence of macrocalcification is generally associated with an increased risk of malignancy (5,25,29). Additionally, it can be difficult to distinguish microcalcification from colloid crystal in the absence of a comet-tail artifact; this prevents the identification of colloid crystal, which typically correlates with benign nodules (5,29,32). In uncertain cases, it is appropriate to use the term “hyperechoic spot”; this feature may be associated with malignancy, as observed in this study and also in other previous reports (31).

A non-ovoid or nonparallel shape (*i.e.*, a tall nodule) was also associated with the likelihood of malignancy in this study, which is consistent with previous reports (8,9,21,25,30). Furthermore, the relationship between height and longitudinal measurement, in addition to transverse measurement, was useful in this analysis. However, the taller-than-wide shape did not exhibit the same degree of association with malignancy compared with other ultrasound features proposed by Kim and cols. (30), a finding that is consistent with that reported by Russ and cols. (10).

We included Doppler flow analysis in this proposed TI-RADS, as done in other studies (7,10,17). Previously, the detection of any degree of internal blood flow was positively related to an increased likelihood of malignancy (17,28,33). However, in our study, this finding was not a useful predictor of malignancy. Only predominant central blood flow was found to be an independent factor associated with the likelihood of malignancy. Similar results regarding the vascularity of thyroid nodules have been reported (26).

In our sample, the presence of blurred margin was identified as an independent factor for benignity, as previously reported, based on its association with Hashimoto’s thyroiditis and benign nodules (31). These results reinforced the idea that a high number of pseudo-nodules in patients with Hashimoto’s thyroiditis may have been aspirated in the control group. Unlike blurred margins, irregular/microlobulated margins were found to be an important feature related to the likelihood of malignancy, which is consistent with findings of previous studies (3-12,21;25-30).

Most suspicious features were not present in a single nodule; conversely, benign and malignant features may overlap (29). All features positively and negatively associated with the likelihood of malignancy – which may be present in the same nodule – should be evaluated to yield an overall score. Previous authors have also evaluated the benign features of thyroid nodules (7,8,10,12,17). However, we attributed different weights to benign and malignant features, which resulted in a new and unique score, unlike the risk score for malignancy created by Kwak and cols. (21). Therefore, a separate evaluation of the findings, as done in prior studies (9,21), is a reliable and better way to predict malignancy than growth rate alone (24), in long-term follow up of thyroid nodules. In this study, as well as in others (9,21,29), a combination of suspicious findings increased the likelihood of malignancy. Moreover, a single feature with a high OR has been found to correlate more strongly with the likelihood of malignancy compared with the manifestation of two minor features (9). Likewise, the presence of features less related to malignancy should not be overlooked. In light of these considerations and our results, spongiform nodules, in the absence of other suspicious features, should not require FNAB. These nodules are associated with a very low risk for malignancy, as previously demonstrated by other researchers (7,25,27,29).

A limitation of this study was the inclusion of limited Bethesda categories since we only evaluated thyroid nodules classified as Bethesda II, V, or VI. The selection criteria based on cytopathology may also have led to the exclusion of follicular carcinomas from our analyses since cytopathology alone is unable to confirm this diagnosis. Even so, a predominantly central flow was a relevant suspicious feature in our scoring system and is a useful predictor of malignancy in follicular neoplasms (5,34,35). In addition, papillary carcinomas are currently the most prevalent differentiated thyroid carcinomas (4), and cytopathology remains the most important tool in the decision to refer patients to surgery.

We did not include elastography in our analysis, which may also be a limitation of this study. However, elastography was also not included in several prior classifications (7-9,11,17), or in the latest ATA guidelines (4).

An additional limitation of this study was the low rate of histopathological confirmation among nodules characterized as benign on cytopathology. However, this limitation has also plagued previous studies for ethical reasons (7-12,17,18). In contrast, our subanalysis including only control thyroid nodules with a confirmed histopathology or a second FNAB strengthened our results. Nodules with two benign cytopathological results are associated with a 100% chance of benignity, as previously reported (4,24).

Important strengths of our study include the fact that all examinations were conducted by a single radiologist, as reported in a previous study (10). Moreover, we indirectly assessed reproducibility by analyzing the agreement between two ultrasound specialists in a subgroup of randomly selected nodules.

In conclusion, this newly proposed TI-RADS involves the quantification of ultrasound features positively and negatively associated with malignancy, with different values attributed to each of these features. We reported the likelihood of malignancy based on cytopathology for different categories of the classification and achieved an adequate association. Additional studies are necessary to validate our findings.
